# 

**Published:** 1893-08

**Authors:** 


### LITERARY.

How Nature Cures: Comprising a New System of Hygiene; also, The Natural Food of Man. By Robert Densmore, M. D. London: Swan, Sonnenschein & Co.; New York: Stillman & Co. pp. 405.

Dr. Densmore's pen is one of the raciest of the profession, and what he writes is always sound logic. In the above book he advances the same lines of thought that have always been put forth by this Journal since its inception.

How to doctor, how to get well and keep well, is, as he says, "nature's engineering," and he uses many very apt and sensible illustrations. The book itself is a family physician, and should find a place in every household in the land.

Speeches of Sir Henry Maine; with a Memoir of his life. By the Right Hon. Sir M. E. Grant Duff. New York: Henry Holt & Co. pp. 451. Price $3.50.

Sir Henry Maine was, beyond a doubt, the recognized leader in the field of jurisprudence. The portrayal of his life, so ably and interestingly pictured by his colleague, is well worthy of perusal. Born of humble parentage, he arose to eminence. He was a professor of civil law at Trinity College, Cambridge, at 23 years. Many personal reminiscences are put forth by the author.

The Popular Science Monthly takes a wide range over the field of science in the July number. It opens with an account, by Henry C. Lea, of the treatment formerly given in Spain to insane offenders against the church, under the title, "The Spanish Inquisition as an Alienist." The views of Herbert Spencer on "Private Relief of the Poor," are given in his well known clear and incisive manner. Dr. W. D. Eastlake enables us to look in again upon daily life in Japan, through a fully illustrated sketch of the "Moral Life of the Japanese." Professor E. S. Tillman describes, also with illustrations. the strange "Fossil Forests of the Yellowstone." Under the title "Are there Evidences of Man in the Glacial Gravels?" the director of the Geological Survey, Major J. W. Powell, defends the action of his assistants in attacking Professor Wright's book on the glacial period; and this controversy receives attention also in the Editor's Table. The number is very interesting throughout.

Lippincott's Magazine for August, contains a complete novel by Robert Barr (Luke Sharp). entitled "In the Midst of Alarms." It is a tale of the Fenian invasion of Canada in 1871. The sixth in the series of the magazine's notable stories is "James Holiday," by Valerie Hays Berry, illustrated. In "The Lady of the Lake," Julian Hawthorne describes some of the statuary and other attractions of the Columbian Exposition.

The athletic series is continued in an article on the national game by Morton B. Young, with portraits of leading players. "Zachary Taylor, His Home and

Family," is by the President's grand-niece, Mrs. Annah R. Watson. W. H. Babcock discusses "Supermundane Fiction," and M. Crofton, in "Men of the Day," presents brief sketches of Sir J. E. Millais, Sir Arthur Sullivan, General Diaz and Philip D. Armour.

THE COSMOPOLITAN for July astonishes the world by its announcement of reduction of its price, twelve and one-half cents per month. The publisher's expectations as to increase in circulation has become fully realized. By reducing its price the general standard of the periodical remains the same—good and substantial. Charles De Kay discusses "A Turning Point in the Arts," illustrated. "The Great Railway Systems of the United States," with its illustrations, is a prominent feature of the number, and is very interesting. William Dean Howells finds time to contribute "A Traveller from Altruria," one of his characteristic pen illustrations. Many other articles appear worthy of careful perusal.

THE CENTURY comes with its usual supply of good things. The July number opens with a glance at a feature of the World's Fair, under the title of "Color in the Court of Honor at the Fair," illustrated; Royal Cortissoz is the contributor. The continued story, entitled "The White Islands," by Mary Hartwell Catherwood, is one of the features. Allan McLane Hamilton writes intelligently upon the subject "Mental Medicine," a remarkable portrayal of treatment of disease by suggestion. There are many other good things that make the number attractive.

The July REVIEW OF REVIEWS opens with a frontispiece of Thomas A. Edison. Under the heading, "Progress of the World," the periodical is replete in its treatment of timely topics of interest to the whole world.

The World's Fair receives considerable attention. Electricity claims a large share of notice. Charles D. Lanier writes interestingly upon "Two Giants of the Electric Age," meaning Thomas A. Edison, while J. Munro also writes jointly upon the work of Sir William Thomson (Lord Kelvin).

Our patriotic exchange, the BLUE AND GRAY, for July, is, as usual, interesting; especially so as we have no publication of its character. Revival of the war is vividly portrayed in its columns—articles written by men who were there and came out to tell the story.

It has just entered upon its second volume, and congratulations are in order.

THE BOOK OF THE FAIR, now in course of publication, says: "A certain engineer, employed by the government in the opening years of the present century on a survey of the great lakes, reported that there was only one spot on the shore of Lake Michigan where a city could not be built. On that very spot the business quarter of Chicago now stands."

THE PRESENT STATUS OF ELECTROLYSIS in the Treatment of Urethral Strictures, a paper by Robert Newman, M. D., of New York, in pamphlet form, has come to our office, and is well worthy of perusal.

"SUMMER DISEASES," is the title of a handy little booklet published by H. K Mulford Co, of Philadelphia.

